# Estimating the Number of Vulnerable People in the United States Exposed to Residential Wood Smoke

**DOI:** 10.1289/ehp.1409136

**Published:** 2015-02-01

**Authors:** Curtis W. Noonan, Tony J. Ward, Erin O. Semmens

**Affiliations:** Center for Environmental Health Sciences, University of Montana, Missoula, Montana, USA

Rogalsky et al. (2014) recently estimated the number of homes and individuals at risk of adverse health effects from exposure to emissions from residential wood combustion in the United States. We appreciate the importance of this topic, particularly to rural and underserved communities. We also understand the authors’ emphasis on low-income individuals because this population generally has more difficulty accessing health care services and fewer resources available to improve indoor air quality. However, several factors suggest that the indication of 500,000–600,000 low-income persons exposed to household air pollution (HAP) from the burning of solid fuels may be a very conservative estimate, substantially underestimating the public health importance of residential wood combustion.

First, the estimate of Rogalsky et al. (2014) was limited by their use of the census-based figure of 2.8 million homes using wood as a primary heating fuel. The U.S. Energy Information Agency (2012) noted that another 8.8 million homes use wood stoves or wood-burning fireplaces as a secondary source of heating. Limited data are available on the frequency of use of wood burning as a secondary heating source and the associated exposure to indoor particulate matter (PM), but the 11.6 million homes with an estimated 2.58 persons per household (U.S. Census Bureau 2012) suggest that closer to 30 million people in the United States live in a home where wood burning is used for heating, rather than the 6.5 million people reported by Rogalsky et al.

Second, the authors’ estimate of at-risk persons was limited to those with co-occurrence of in-home wood burning as a primary heating source and households below the Federal Poverty Level (FPL) (i.e., 900,000 people that meet both criteria). However, at-risk individuals, including children and the elderly, also reside in homes that are above the FPL threshold. Third, Rogalsky et al. suggested that 53–65% of wood-burning homes in high-poverty communities may exceed health-based standards, but this estimate is based on few studies, with poverty assessed only at the community level. Finally, the authors focused only on direct indoor fugitive emissions in homes using wood stoves, but exposure risk is not limited to those living in homes with wood-burning appliances. As indicated in several published studies, communities with a high proportion of residential wood-burning households may also have elevated concentrations of ambient wintertime PM (Ward and Lange 2010). Moreover, analyses of infiltration efficiencies suggest that exhausted wood smoke can contribute substantially to indoor PM concentrations in both wood-burning and non–wood-burning homes (Barn et al. 2008), resulting in a higher proportion of homes and their residents experiencing risk from biomass combustion–derived PM. Rogalsky et al. (2014) should be commended for acknowledging these and other limitations in their discussion, and we appreciate the opportunity to provide additional information on these points.

Here we offer an alternative framework for estimating the number of people in the United States exposed to high levels of PM associated with wood burning. Approximately 11.6 million homes in the United States use wood as a primary or secondary source of heat. Of these, 4.8 million homes have wood stove appliances (U.S. Energy Information Agency 2012). Because of the uncertain frequency of fireplace use, we have not included homes with fireplaces in our estimate, although they likely are important sources of indoor PM. Rather than limiting our estimate to those homes below the FPL, we define our at-risk population as the susceptible individuals living within these homes (i.e., children and the elderly). With approximately 0.63 children < 18 years of age per household and 0.33 people > 65 years of age per household (U.S. Census Bureau 2012), we estimate that within the United States alone, approximately 4.8 million susceptible individuals live in homes with substantial exposures to wood smoke–derived PM, an order of magnitude greater than the 0.5–0.6 million estimate of Rogalsky et al. (2014). This estimate is conservative because it does not account for infiltration into non–wood stove households experiencing HAP generated from neighboring wood-burning homes, nor does it account for all household residents that are vulnerable due to chronic health conditions.

As with any estimates of at-risk populations, there is an important balance to strike between underestimating the risk and artificially inflating the public health importance. We suggest that Rogalsky et al. (2014) erred toward the former. Our estimates are based on a different framework with respect to exposure potential and susceptible populations. Whether the true number of individuals in the United States at risk for adverse health effects from exposure to wood smoke is closer to 0.5 million or 4.8 million, it remains clear that this is an important environmental exposure that disproportionately impacts rural populations.
